# Pressure Dependence of Structural Behavior in the Polymorphs of Fe(PM–BiA)_2_(NCS)_2_

**DOI:** 10.3390/molecules30122651

**Published:** 2025-06-19

**Authors:** Pulkit Prakash, Hend Shahed, Ji Qi, Andrzej Grzechnik, Manuel Angst, Jörg Voigt, Jörg Perßon, Yao Cheng, Biliana Gasharova, Yves-Laurent Mathis, Francesco Capitani, Carsten Paulmann, Charlie McMonagle, Dmitry Chernyshov, Karen Friese

**Affiliations:** 1 Jülich Center for Neutron Science—2, Forschungszentrum Jülich GmbH, 52425 Jülich, Germany; p.prakash@fz-juelich.de (P.P.); h.shahed@fz-juelich.de (H.S.); ji.qi@fz-juelich.de (J.Q.); m.angst@fz-juelich.de (M.A.); j.voigt@fz-juelich.de (J.V.); jo.persson@fz-juelich.de (J.P.); 2 Jülich Center for Neutron Science—4, Forschungszentrum Jülich GmbH, 52425 Jülich, Germany; a.grzechnik@fz-juelich.de; 3 Institut für Kristallographie, RWTH Aachen University, Jägerstr. 17-19, 52066 Aachen, Germany; cheng_yao@hotmail.com; 4 Institute for Beam Physics and Technology, Karlsruhe Institute of Technology, 76021 Karlsruhe, Germany; biliana.gasharova@kit.edu (B.G.); yves-laurent.mathis@kit.edu (Y.-L.M.); 5 Synchrotron SOLEIL, L’Orme des Merisiers, Saint-Aubin, 91192 Gif-sur-Yvette, France; francesco.capitani@synchrotron-soleil.fr; 6 Mineralogisch-Petrographisches Institut, Universität Hamburg, Grindelallee 48, 20146 Hamburg, Germany; carsten.paulmann@desy.de; 7 Swiss-Norwegian Beamlines, European Synchrotron Radiation Facility, 38000 Grenoble, France; charles.mcmonagle@esrf.fr (C.M.); dmitry.chernyshov@esrf.fr (D.C.)

**Keywords:** high-pressure studies, spin-crossover compounds, spectroscopy

## Abstract

The pressure dependence of structural behavior in the orthorhombic (*Pccn*, PI) and monoclinic (*P2*_1_/*c*, PII) polymorphs of the compound [Fe(PM-BiA)_2_(NCS)_2_], where PM–BiA = (N–(2′–pyridylmethylene)–4-amino–bi–pheynyl), is studied with synchrotron single-crystal X-ray diffraction and vibrational spectroscopy. Both polymorphs are stable up to ∼1.5 GPa, with a spin state transition occurring only in polymorph PII under hydrostatic conditions as documented by single-crystal synchrotron diffraction. The diffraction data also provide evidence of the formation of superstructures for both PI, with a doubled *c* axis, and PII, with a doubled *b* axis, on applying pressures above 2 GPa. The LS and HS states seem to coexist at high-pressures for both polymorphs studied with synchrotron infrared spectroscopy at quasi-hydrostatic conditions. Such results indicate that the occurrence of spin-crossover transformations in [Fe(PM-BiA)_2_(NCS)_2_] might strongly depend on the stress in the sample.

## 1. Introduction

The search for novel caloric materials, which can be utilized as refrigerants in the new generation of solid-state refrigerators with improved energy efficiency and less environmental impact [1], is gaining continuous momentum [2,3,4,5]. Recently, the family of iron(II) spin-crossover compounds has been discussed as a potential candidate based on their substantial isothermal entropy (ΔS_*T*_) and adiabatic temperature (ΔT_*ad*_) changes associated with the spin-state transition from the low-spin (LS) (S = 0, $t_{2g}^{6}$ $e_{g}^{0}$) to the high-spin (HS) (S = 2, $t_{2g}^{4}$ $e_{g}^{2}$) state [6,7]. The spin-state transition primarily originates from the competing crystal field and electron pairing energies around the central metal ion, among which the former can be tuned by using several external perturbations such as temperature [8,9], pressure [10], light irradiation [11,12], magnetic [13] and electric fields [14], and guest molecules [15,16]. This tunability allows for multiple applications [17,18].

In the spin-crossover (SCO) transition from the HS to the LS state, a redistribution of the electron density on the bonding (t_2*g*_) orbitals occurs, which leads to a shortening of the metal–ligand bond distances [19]. This change at the molecular level is then transmitted across the lattice via intermolecular contacts, such as hydrogen bridges and *π*–*π* interactions [20]. Therefore, the electronic re-configuration couples strongly with the lattice and other physical properties of the system [8]. From a thermodynamic perspective, the spin-crossover is driven by a competition between the enthalpy, which favors the low-spin state present at low temperatures and high pressures, and the entropy, which favors the high-spin state present at high temperatures and low pressures [21].

The nature of the transition between the HS and LS states can be abrupt or gradual [20,22]. However, the prediction of the nature of SCO transition based solely on the nature of ligands and the packing of the crystal structure remains impossible [21]. Therefore, apart from the ongoing search for suitable materials for barocaloric applications, the investigation of spin-crossover transitions is also driven from a fundamental physics perspective to better understand the complex nature of interactions governing the physical properties and the nature of the spin transition of such systems [23].

**Figure 1:**
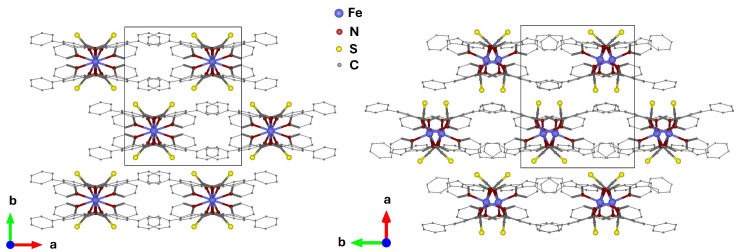
Schematic drawing of the molecules and crystal structures of the orthorhombic (PI, *Pccn*) (**left panel**) and monoclinic (PII, *P2*_1_/*c*) (**right panel**) polymorphs, depicting the layered structure of the molecules.

We consider the well-known compound [Fe(PM–BiA)_2_(NCS)_2_], where PM–BiA = (N–(2′–pyridylme-thylene)–4-amino–bi–pheynyl) [20,24] crystallizes in two different polymorphs with significantly different characteristics of the spin-crossover transitions. In the orthorhombic polymorph PI (space group *Pccn*) the temperature-induced spin transition is abrupt (T_1/2_ = 177 K; ΔT = 1 K), while in the monoclinic polymorph PII (space group *P2*_1_/*c*) the spin state transition is gradual (T_1/2_ = 210 K; ΔT = 100 K), where ΔT denotes the temperature range of the compound’s mixed spin state (both spin states > 10%), and T_1/2_ is the temperature at which HS and LS states are equally populated. The structures are described as being composed of molecular slabs, which extend in the (*a,c*) plane with *b* as the stacking direction in PI, while the planes extend in the (*b,c*) plane and are stacked along *a* in PII (Figure 1). As the molecular units in both polymorphs are basically identical, they represent an ideal system to study the role of intermolecular interactions and their relationship to the underlying dynamics. In this study, we do not provide a direct assessment of the barocaloric potential of the compound; however, the pressure dependence of structural and spin-state transitions is critical for the barocaloric effect, and the correlation of temperature and pressure dependencies should thus provide insight for identifying promising barocaloric candidates.

While detailed temperature-dependent single-crystal investigations were already carried out on both polymorphs [20,24,25] (and also on the closely related [Fe(PM–PeA)_2_(NCSe)_2_], [26]), only a limited number of experimental [10,27,28,29] and theoretical [30,31] studies were carried out under pressure. These studies suggest [27] that PI undergoes a structural phase transition to polymorph PII at a pressure of ∼0.75 GPa. It should be noted, however, that these experimental studies were performed on polycrystalline samples, and the data were not sufficient to follow the structural evolution as a function of pressure.

In this work, we present a comprehensive high-pressure investigation, utilizing synchrotron single-crystal X-ray diffraction, as well as infrared and Raman spectroscopy, for the PI and PII polymorphs of [Fe(PM–BiA)_2_(NCS)_2_]. Based on our data, we (i) examine the correlation between structural changes and phonon modes during the spin-state transitions as well as (ii) elucidate the pressure-induced phases.

## 2. Results and Discussion

### 2.1. *Synchrotron X-Ray Diffraction*

**Figure 2:**
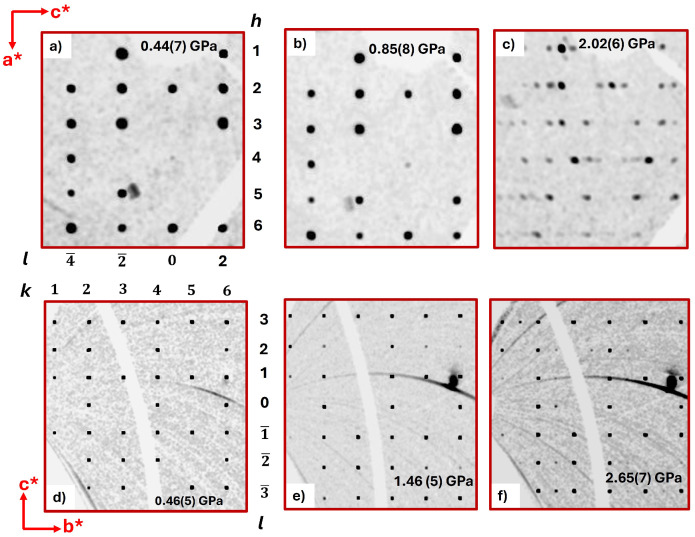
Reconstructed reciprocal space sections: (**top**) (*h0l*) plane for the orthorhombic (PI, *Pccn*) polymorph at (**a**) 0.44(7) GPa, (**b**) 0.85(8) GPa, and (**c**) 2.02(6) GPa and (**bottom**) (*0kl*) plane for the monoclinic (PII, *P2*_1_/*c*) polymorph at (**d**) 0.46(5) GPa, (**e**) 1.46(5) GPa, and (**f**) 2.65(7) GPa.

Reconstructions of reciprocal space of both polymorphs and subsequent refinements of the structures show that polymorphs PI and PII remain stable up to 1.36 GPa and 1.46 GPa, respectively (Figure 2; Tables S1 and S2 in the Supplementary Materials). With a further increase in pressure, superstructure reflections appear in both polymorphs (starting from 2.02(6) GPa (PI) and 2.65(7) GPa (PII)). For polymorph PI, they indicate a doubling of the *c* axis, while for polymorph PII, they indicate a doubling of the *b* axis (Figure 2). In both superstructures, the doubling of the lattice parameters occurs within the molecular slabs, albeit in different directions (Figure 2). Refinements of the superstructures using models derived from the lower pressure structures were not successful, indicating that there are substantial structural rearrangements. Several attempts to solve the superstructures using Direct Methods, Charge Flipping, and Patterson Methods did not lead to a satisfactory structure solution either. We attribute this to the low completeness of the data.

**Figure 3:**
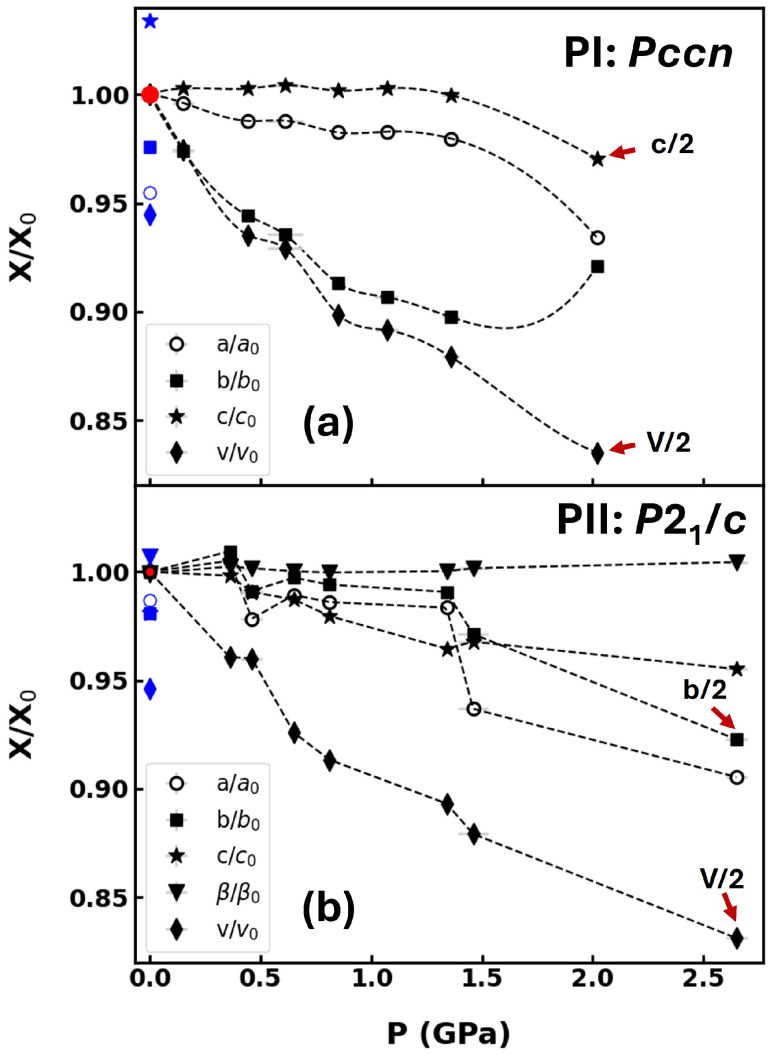
Pressure dependence of the normalized unit-cell parameters and volume of the orthorhombic (PI, *Pccn*) (**a**) and monoclinic (PII, *P2*_1_/*c*) polymorph (**b**) at 300 K. Data points at 2.02 and 2.64 GPa correspond to the superstructures. Red and blue symbols represent values in the HS (PI: 300 K; PII: 270 K) and in the LS state (PI: 95 K; PII: 93K) at ambient pressure [20]. Lines are guides to the eyes. The lattice parameters and unit-cell volumes indicated with red arrows at highest pressures are scaled with a factor of (1/2). Error bars are smaller than symbols.

Both polymorphs exhibited a decrease in unit-cell volumes with comparable relative changes dV/dP for polymorphs PI and PII up to 1.36 and 1.46 GPa, respectively (Figure 3). No indication of a volume expansion in PI at 0.7 GPa, as reported in earlier studies [28], was observed. Both polymorphs exhibited strong anisotropic compressibility. The largest compressibility occurred along the stacking directions of the molecular planes (*b* and *a* axes for PI and PII, respectively). Due to the significant problems in collecting consistent high-pressure diffraction data on different beamlines and the resulting limited number of data points, a fit of the unit cell volume with an equations of state [32] was not attempted.

**Figure 4:**
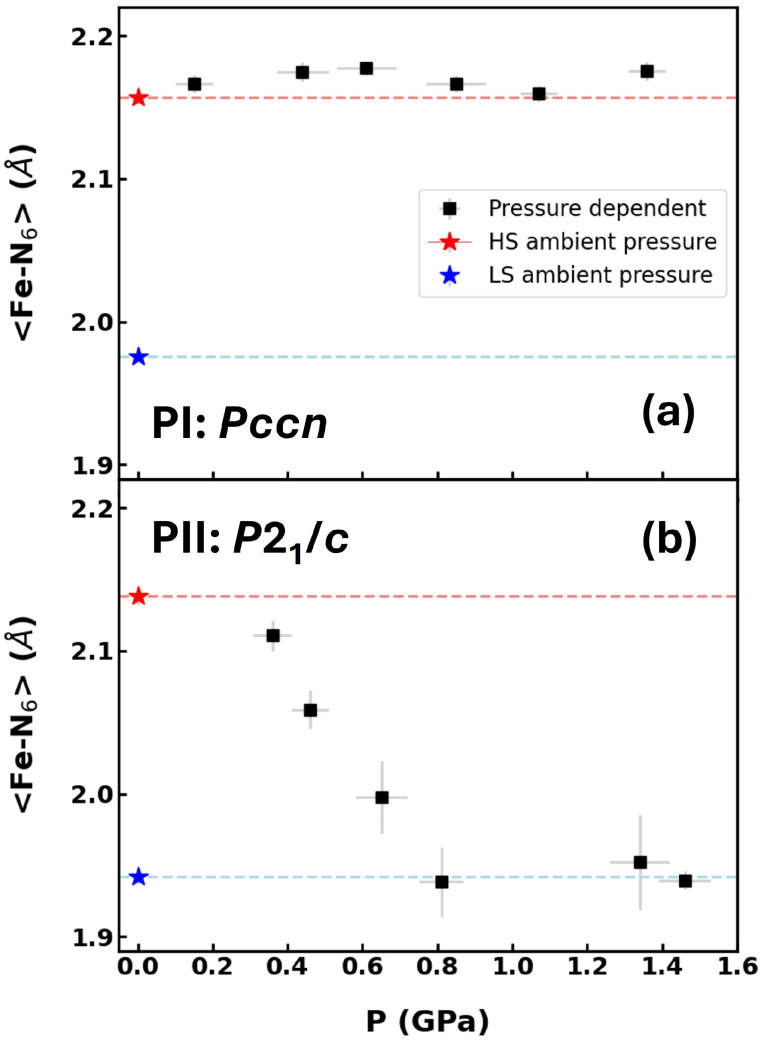
The change of the average $\langle \mathrm{Fe}-N_{6}\rangle$ bond length for the (**a**) orthorhombic (PI, *Pccn*) and (**b**) and monoclinic (PII, *P2*_1_/*c*) polymorphs, as a function of pressure at ambient temperature. The values in the HS and LS state at ambient pressure are shown as red and blue stars, respectively. They are extrapolated to high pressures as dotted lines.

An inspection of the pressure evolution of the averaged octahedral $\langle \mathrm{Fe}-N_{6}\rangle$ bond length, as obtained from the single-crystal structure refinements, shows that for polymorph PI, the value remained approximately constant up to 1.4 GPa, while for polymorph PII, it monotonously decreased up to 0.8 GPa, where it reached the value corresponding to the low-spin state at ambient conditions and then became constant (Figure 4). This implies that there is no pressure-induced spin-crossover transition in PI at pressures up to 1.36 GPa, even though the unit-cell volumes on compression were lower than the one where the LS state was formed at low temperatures and atmospheric pressure. Earlier powder neutron diffraction [27,28] and first principles calculations [30] had suggested a phase transition to polymorph PII at about 0.75 GPa at different temperatures including room temperature. At least at room temperature, such a transition can be ruled out by our experiments. For PII, a gradual pressure-induced spin-crossover transition occurred on compression to 0.8 GPa. Previously, the HS state in PII was found to be present to 0.135 GPa using reflectance studies [29], which is consistent with our result only starting at a higher pressure.

### 2.2. *Vibrational Spectroscopy*

**Figure 5:**
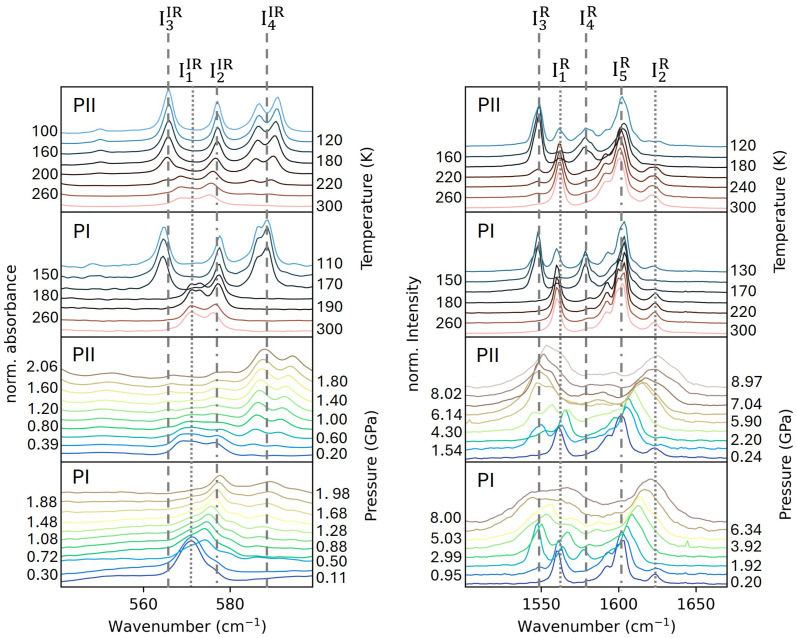
(**left**) Comparison of the temperature (KIT) and pressure (SOLEIL) evolution of Fe-N vibrational phonon modes, obtained from the infrared spectra, normalized to the peak height at 670 cm^−1^. Data are offset vertically for clarity. (**right**) Comparison of the temperature and pressure evolution of amine stretching vibrational modes obtained from the Raman spectra. Dashed lines point to features in the LS state; dotted lines point to features in the HS state; dashed dotted lines assign peaks present in both states.

The left panel in Figure 5 shows a comparison of the temperature and pressure evolution (up to about 2 GPa) of infrared modes of the $\langle \mathrm{Fe}-N_{6}\rangle$ unit in both polymorphs in the wavenumber range from 545 to 600 cm^−1^ [33,34]. In this wavenumber range, the spectra for both polymorphs are very similar because the coordination of the Fe atoms to the N atoms is quite alike. In the spectra measured as a function of temperature at ambient pressure, we observed modes $I_{1}^{\mathrm{IR}}$ and $I_{2}^{\mathrm{IR}}$ in the HS state. In the LS state, the $I_{1}^{\mathrm{IR}}$ mode vanished while additional modes $I_{3}^{\mathrm{IR}}$ and $I_{4}^{\mathrm{IR}}$ appeared. The $I_{4}^{\mathrm{IR}}$ doublet is due to the factor group splitting [35]. The magnitude of this splitting could be considered as a measure of the influence that the crystal lattice exerts on the molecule. It is larger in the monoclinic PII polymorph than in the higher symmetry one PI. On cooling, the $I_{2}^{\mathrm{IR}}$ mode shifts to higher energies but still exists in the LS state. Altogether, Figure 5 reveals that at atmospheric pressure a sharp SCO transition in PI occurred between 180 and 170 K, while in PII, we observed a coexistence of low LS and HS features in an extended temperature region of 160 K < T < 230 K.

Part of the differences between the temperature- and pressure-dependent spectra, collected from SOLEIL and KIT light sources shown in Figure 5, arises from the different spectral resolutions utilized at the two facilities and the dependence of the spectral intensities on the crystal orientation. Apart from that, the CsI pressure-transmitting medium used in the pressure-dependent measurements is not exactly hydrostatic. It induces broadening of the observed bands due to deviatoric stress in the samples [36].

A comparison with the pressure evolution of the phonon modes in the same wavenumber range indicates that the onset of a gradual transition to the LS state in PII occurs at about 0.4 GPa with the appearance of the $I_{4}^{\mathrm{IR}}$ modes. The mode $I_{3}^{\mathrm{IR}}$ with very low intensity could be observed above 0.6 GPa. A weak spectral feature $I_{1}^{\mathrm{IR}}$ of the HS state could still be traced up to 2 GPa. The persistence of a small phase fraction of the HS state at 2 GPa may be attributed to short-range correlations of the HS state, which compete with the dominant LS matrix, as reported in the literature for other compounds [37]. The IR data for PII thus corroborate the structural data, depicting a smooth gradual transition with increasing pressure.

In PI, mode $I_{4}^{\mathrm{IR}}$, which is associated with the LS state, started to appear at about 0.5 GPa. Both the HS and LS states seem to have coexisted up to 2 GPa. The fact that both were observed in the spectroscopic data but not in the diffraction data could be explained with the use of CsI as a pressure-transmitting medium in IR experiments. On the other hand, the absence of the $I_{3}^{\mathrm{IR}}$ mode (Figure 5) also suggests that the emergence of the $I_{4}^{\mathrm{IR}}$ mode could be correlated with the formation of the superstructure detected in the X-ray data. In this case, the superstructures would therefore happen in different spin states: HS for PI and LS for PII. The presence of only the HS state for PI in the low-pressure structure, with moderately increasing pressure at 300 K, would be consistent with the literature, including high-pressure magnetization [10] and diffuse reflectance measurements [29].

The vibrational modes that exhibit the maximum intensity in the Raman spectra are associated with the α-dimine stretching vibrations [38]. We identified the modes $I_{3}^{R}$ and $I_{4}^{R}$ as markers for the LS state Figure 5. At ambient pressure and room temperature, the spectra look very similar for both polymorphs. Upon cooling down to the LS state, the characteristic features of the HS state re-appeared. We attribute it to the light-induced excited state spin-trapping effect (LIESST) [39,40] induced by the blue laser (λ = 488 nm) utilized during the measurements. Similar effects were also observed in the low-spin state of other compounds, in particular if they exhibited intermolecular interactions such as π–π interactions [41]. The highest temperature at which LIESST can be observed depends on the scan rate (T_LIESST_) [42]. In our study, the use of a scan rate of 10 K min^−1^ shifted T_LIESST_ to higher values, resulting in the formation of mixed HS–LS states at the lowest measured temperatures of 130 K and 100 K for PI and PII, respectively. The potential local heating of the sample by the laser, which could lead to a thermally induced transition rather than a purely photonic one, might also be related to this observation [43].

It might be possible that with further lowering of the temperature, a complete low-spin state could occur in both the polymorphs. However, measurements at lower temperatures were prevented by the thermosalient behavior [44,45] at approximately ≤100 K that made it impossible to record the spectra. The pressure dependence of the α-diimine stretching Raman-active vibrations for both polymorphs is also shown in Figure 5. In this experiment, we probed a wider pressure range up to 5.9 GPa. To about 0.2 GPa, the spectra of both polymorphs changed very little. At higher pressures, the additional bands $I_{3}^{R}$ and $I_{4}^{R}$ appeared in PI as well as $I_{3}^{R}$ in PII. The bands broadened on compression, precluding any conclusive band assignment.

## 3. Materials and Methods

### 3.1. *Single-Crystal X-Ray Diffraction*

High-pressure single-crystal diffraction measurements were performed on a kappa diffractometer at beamline P24 (λ = 0.413(3) Å, PETRA III, Hamburg, Germany) [46] and on the one-circle multipurpose diffractometer at the BM01 beamline of the Swiss–Norwegian Beamlines (λ = 0.720(4) Å, SNBL, ESRF, Grenoble, France) [47]. The beamlines are equipped with a Pilatus 1M CdTe and a Pilatus 2M detector (Dectris, Baden, Switzerland), respectively. A Boehler–Almax-type diamond anvil cell (DAC) [48] was used at the P24 beamline for both polymorphs, while at the SNBL, a Yao-DAC cell [49,50] (see Figure S1 in the supplementary materials) with a wide opening angle of 120° was utilized for PI and a Boehler–Almax DAC for PII. Isopropanol was used as the pressure-transmitting medium. The pressure was determined with the ruby luminescence method [51,52]. It is noteworthy that the crystals frequently broke while loading the DAC; however, data were collected in single runs on individual crystals.

Data analysis and integration were performed with the CrysAlis PRO software (Version 1.171.37.33) package [53]. Structures at ambient conditions [20] were used as starting models for the refinements using *JANA2006* [54]. Due to the low completeness of the data (30%), displacement parameters were modeled only isotropically. For the monoclinic polymorph, the atomic displacement parameters were constrained to be identical for (a) sulfur atoms and (b) carbon atoms within one aromatic ring. Hydrogen atom positions were calculated using the riding model with C–H bond distances set to 0.96 Å.

### 3.2. *Infrared Spectroscopy*

Temperature dependent infrared spectroscopic measurements were conducted at the IR2 beamline of the KIT light source (Karlsruhe Institute of Technology, Karlsruhe, Germany). Spectra in the range 300–4000 cm^−1^ were acquired using a Vertex80v FTIR spectrometer coupled to an IRscopeII (Bruker, Billerica, MA, USA) microscope. Measurements were performed in transmitted-light mode using Schwarzschild objectives (Thermo Oriel, Stratford, CT, USA) (15×, 0.4 N.A.). Far infrared spectra were recorded with a liquid He cooled bolometer detector and a broadband Mylar beamsplitter (Bruker Optik GmbH, Ettlingen, Germany) whereas a liquid N_2_ cooled MCT (HgCdTe) [55] detector and a KBr beamsplitter were used for the mid-infrared region. Single crystals of the orthorhombic and monoclinic polymorphs (∼70 × 70 × 50 μm^3^) were placed on a KBr pellet and loaded in a LINKAM FTIR600 stage equipped with KBr lid windows (Linkam scientific instruments Ltd., Salfords, UK). Prior to the measurements, the stage was purged with dry N_2_ gas to remove humidity.

The infrared spectra were measured over the temperature range of 80–300 K in steps of 20 K in both cooling and warming cycles, with smaller steps (5 K) in the vicinity of the spin-crossover temperatures. The rate of change of temperature was kept at 10 K/min with an equilibration time of 3 min at each temperature. The spectra were recorded with 128 accumulations (each of 1 s) with a spectral resolution of 1 cm^−1^. Background spectra were recorded through the empty KBr sample holder prior to every sample measurement and were subsequently used for the calculation of the absorbance data. The positions of the IR bands were determined from the experimental data using the software OPUS v8.5 [56].

High-pressure infrared spectra up to 2 GPa were recorded at the SMIS beamline [57] of the synchrotron SOLEIL (Saint-Aubin, France) using a homemade horizontal microscope with custom Schwarzschild objectives (N.A. = 0.5). Two membrane-driven diamond anvil cells, having type-IIa diamonds with culets of 600 μm and 800 μm, were utilized for collecting the high-pressure data of PI and PII, respectively. Pressure was determined with the ruby luminescence method [51,52]. Finely ground CsI powder served as pressure-transmitting media, making it possible to collect the transmittance data in the broad spectral range of 150–10,000 cm^−1^ by a Thermo-Fisher iS50 interferometer (Waltham, MA, USA) with KBr and solid substrate beamsplitters, using a MCT detector and liquid helium cooled bolometer. The data were collected in steps of 0.1 GPa for PI and 0.2 GPa for PII at T = 300 K. The spectra were recorded with 128 accumulations, with a spectral resolution of 2 cm^−1^. The background was recorded on bare CsI inside the DAC and subsequently used for the calculation of the absorbance data. The positions of the IR bands were determined with the software OMNIC v8.2 [58].

### 3.3. *Raman Spectroscopy*

Raman spectra were collected on single crystals of both polymorphs in a backscattering geometry on a WITec alpha300 R micro-Raman spectrometer (WITec Wissenschaftliche Instrumente und Technologie GmbH, Ulm, Germany) coupled to a 488 nm solid state laser in the spectral range 60–2600 cm^−1^ using an edge filter for Rayleigh line rejection, a Nikon 20× (Nikon Instruments Europe B.V., Amsterdam, The Netherlands) (N.A. 0.35) long-working distance objective, a 1800 grooves mm^−1^ grating, and a CCD detector, available at the KIT light source. The laser power was kept at 40 μW during the measurements. The same LINKAM stage that was utilized in the case of IR was used for the Raman experiments. In total, 4 scans with an integration time of 128 s were collected at a spectral resolution of ∼0.9 cm^−1^ with temperature steps of 20 K in the range 80–300 K. The temperature variation and the equilibration time were the same as in the IR measurements. High-pressure Raman spectra at 300 K were recorded in the same setup using Boehler–Almax DAC filled with KBr as the pressure-transmitting medium. The data was recorded in steps of 0.5 GPa, and the pressure was monitored by the ruby luminescence method [51,52]. It should be noted that solid pressurizing media, such as KBr [59,60] and CsI [36], though quasi-hydrostatic in nature, are IR- and Raman-transparent [61], making them suitable candidates for spectroscopic studies. On the other hand, liquid-pressurizing media, like isopropanol [62], though hydrostatic in nature, exhibit numerous vibrational modes that are both IR- and Raman-active [63], which obscure the signal originating from the sample, thus making them unsuitable for spectroscopic investigations.

## 4. Conclusions

In this work, we have employed single-crystal synchrotron diffraction as well as infrared and Raman spectroscopy measurements at extreme conditions in order to shed light on the behavior of the orthorhombic (*Pccn*, PI) and monoclinic (*P2*_1_/*c*, PII) polymorphs of the spin-crossover compound [Fe(PM-BiA)_2_(NCS)_2_]. The vibrational data confirm the previous observations [20] that the SCO in monoclinic PII is gradual, while it is very sharp in orthorhombic PI as a function of temperature at atmospheric conditions.

Our studies demonstrate that with increasing pressure to about 1.5 GPa, both polymorphs remain stable, with a spin-crossover induced in PII but not in PI, as seen with X-ray diffraction, which was performed with an isopropanol hydrostatic pressure medium. We only observe the shortening of the Fe–N bond distances, which is characteristic of SCO [6] in PII but not in PI. To the best of our knowledge, a pressure-induced SCO at 300 K for PII was not previously reported. On the other hand, the LS and HS states are suggested to coexist at high pressures for PI, as documented by the infrared spectroscopy data collected with a CsI quasi-hydrostatic pressure medium. This indicates that the occurrence of SCO in PI might strongly depend on the stress in the sample. Above about 2 GPa, using single-crystal synchrotron diffraction, we observed superstructures which correspond to the doubling of the *c* lattice parameter in polymorph PI and a doubling of the *b* lattice parameter in polymorph PII. The doubled lattice parameters correspond to directions of intra-plane molecular arrangements. Our studies reveal that a compound having gradual SCO with temperature also displays a similar gradual SCO with pressure, suggesting that such materials may be suitable candidates for barocaloric applications, given their continuous response to external stimuli.

A single-crystal diffraction study carried out under uniaxial pressure could help to shed more light on the structural features involved in the pressure-induced SCO. On the other hand, by deuterating the materials, one can study the influence of the H(D)-bonding network and π–π interactions on the SCO process. To address the issue of spin states in the superstructures of both polymorphs, experiments such as Mössbauer spectroscopy or nuclear forward scattering under high pressures could provide valuable insights, answering in particular the question of whether the superstructures involve ordered HS and LS states, as observed in other SCO compounds [64]. Additionally, a detailed DFT calculation on the whole unit cell, at different pressures and taking dispersion forces into account, can lead to a more comprehensive understanding of the different SCO behavior in both polymorphs.

## Data Availability

The data are available upon request.

## Supplementary Materials

The following supporting information can be downloaded at https://www.mdpi.com/article/10.3390/molecules30122651/s1. Figure S1: (left) Yao-DAC for X-ray and neutron single-crystal diffraction. The diamond anvil cell with an opening of 120 (Yao-DAC) is based on the design by Yao Cheng [50]. Its diameter is 40 mm. The body parts as well as the seats are made of the NiCrAl alloy allowing for using it both for X-ray and neutron single-crystal diffraction [49]. The body parts have matching crenel-like cuts. The screws (M4 and M1.6) and the locking ring for the rocker seat are made of titanium (grade 2). Both parallel and translational alignments of the diamonds are possible; Table S1: Selected experimental crystal data for the orthorhombic (PI, *Pccn*) polymorph of [Fe(PM–BiA)_2_(NCS)_2_] at different pressure points; Table S2: Selected experimental crystal data for the monoclinic (PII, *P2*_1_/*c*) polymorph of [Fe(PM–BiA)_2_(NCS)_2_] at different pressure points.
