# Supplementary material for: Pressure Dependence of Structural Behavior in the Polymorphs of Fe(PM–BiA)2(NCS)2
Source: Molecules. 2025 Jun 19;30(12):2651. doi: 10.3390/molecules30122651 (PMC12195809; doi:10.3390/molecules30122651)
Supplement: Supplementary file 1 [file molecules-30-02651-s001.zip › molecules-3676173-supplementary.pdf]

1. Tables

Table S1. Selected experimental crystal data for the orthorhombic (*Pccn*, PI) polymorph of [Fe(PM-BiA)$_2$(NCS)$_2$] at different pressure points.

**Chemical Formula**	**Orthorhombic** $FeN_6S_2C_{38}H_{28}$					
**Pressure(GPa)**	**0.44**	**0.85**	**1.36**	**0.15**	**0.61**	**1.07**
a (Å)	12.7982(13)	12.7285(15)	12.6944(16)	12.9059(5)	12.7999(5)	12.735(3)
b (Å)	14.360(4)	13.881(6)	13.638(6)	14.8090(6)	14.221(6)	13.784(8)
c (Å)	17.650(6)	17.6348(18)	17.597(6)	17.6530(6)	17.680(6)	17.657(5)
V ($Å^3$)	3243.8(2)	3116.1(2)	3046.5(2)	3373.9(2)	3218.2(2)	3099(2)
Density (g cm^{-1})	1.4113	1.4687	1.5027	1.3569	4.9746	5.1652
μ (mm^{-1})	0.147	0.154	0.157	0.615	2.236	2.322
Radiation Type		Synchrotron			Synchrotron	
Wavelength (Å)		0.413(3)			0.720(4)	
Diffractometer		κ-diffractometer using a Pilatus 1M CdTe			Multipurpose PILATUS @ SNBL diffractometer	
No. of measured, symmetry independent, and observed [I > $2\sigma(I)$] reflections	13625	14040	13295	2436	3447	2274
	1899	1855	1812	1057	1346	917
	1034	1116	1050	507	671	400
R_{int}	0.1747	0.1389	0.1845	0.0582	0.0607	0.0863
(sin θ / λ) $(Å)^{-1}$	0.67	0.67	0.67	0.8	0.8	0.8
No. of parameters	113	103	95	213	213	213
R[$F^2 > 2\,\sigma(F^2)$]	0.1631	0.1221	0.1733	0.0557	0.0636	0.0384
wR(F^2)	0.0988	0.0934	0.1425	0.0403	0.0575	0.0275
S	3.3	3.52	4.54	2.1	2.65	1.33

Table S2. Selected experimental crystal data for the monoclinic ($P2_1/c$, PII) polymorph of [Fe(PM-BiA)$_2$(NCS)$_2$] at different pressure points.

Chemical Formula	Monoclinic $FeN_6S_2C_{38}H_{28}$					
**Pressure(GPa)**	**0.36**	**0.65**	**0.81**	**1.34**	**0.46**	**1.46**
a (Å)	16.885(3)	16.700(6)	16.528(2)	16.316(2)	16.867(2)	16.152(2)
b (Å)	12.3716(13)	12.2217(13)	12.1829(17)	12.1419(10)	12.3747(10)	12.1258(10)
c (Å)	17.219(2)	17.6348(18)	17.597(6)	16.848(2)	17.181(2)	16.7840(2)
β (°)	116.120(15)	115.85(15)	116.902(2)	115.86(15)	115.930(15)	115.936(15)
V (Å^3)	3225.8(7)	3111.1(2)	3062.3(2)	3003.5(7)	3225.07(7)	2956.17(7)
Density (g cm^{-1})	1.4183	1.4701	1.4937	1.5229	1.4183	1.5473
μ (mm^{-1})	0.147	0.152	0.155	0.158	0.638	0.695
Radiation Type	Synchrotron			Synchrotron		
Wavelength (Å)	0.413(3)			0.720(4)		
Diffractometer	κ-diffractometer using a Pilatus 1M CdTe			Multipurpose PILATUS @ SNBL diffractometer		
No. of measured, symmetry independent, and observed $[I > 2\sigma(I)]$ reflections	13763<br>3132<br>1317	13772<br>3054<br>1358	12592<br>1515<br>859	1333<br>2917<br>1376	2627<br>1522<br>1071	2608<br>1470<br>1231
R_{int}	0.227	0.2057	0.306	0.2481	0.0694	0.0455
$(\sin\theta / \lambda)$ (Å)$^{-1}$	0.55	0.55	0.55	0.55	0.65	0.68
No. of parameters	153	155	152	155	204	204
R[$F^2 > 2\sigma(F^2)$]	0.2145	0.1727	0.284	0.2078	0.1003	0.0735
wR(F^2)	0.1388	0.1275	0.2305	0.1791	0.1195	0.0867
S	3.43	3.27	5.97	4.19	6.75	5.55

2. Figures

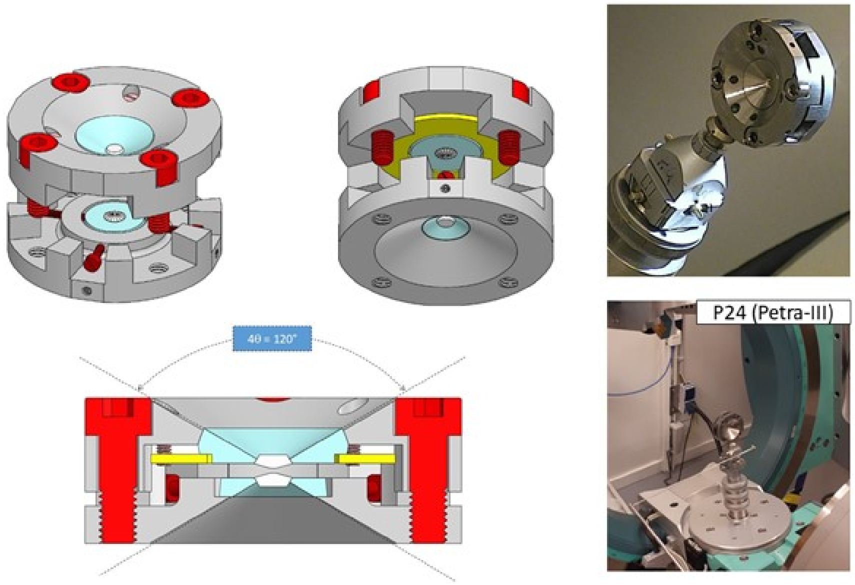

Figure S1. (left) **Yao-DAC for X-ray and neutron single-crystal diffraction**
The diamond anvil cell with an opening of 120° (Yao-DAC) is based on the design by Yao Cheng [1]. Its diameter is 40 mm. The body parts as well as the seats are made of the NiCrAl alloy allowing for using it both for X-ray and neutron single-crystal diffraction [2]. The body parts have matching crenel-like cuts. The screws (M4 and M1.6) and the locking ring for the rocker seat are made of titanium (grade 2). Both parallel and translational alignments of the diamonds are possible.

References

1. Cheng, Y. A Diamond Anvil Cell for Combined X-Ray and Neutron Single- Crystal Studies. Thesis, 2018.
2. Cheng, Y.; Brenk, J.; Friedrich, B.; Perßon, J.; Maraytta, N.; Gibson, J.S.K.L.; Korte-Kerzel, S.; Roth, G.; Su, Y.; Zhu, F.; et al. Ni–Cr–Al Alloy for neutron scattering at high pressures. *Materials Science and Technology* **2020**, *36*, 949–954. https://doi.org/10.1080/02670836.2019.1578077.
